# Association between COVID-19 Booster Vaccination and COVID-19 Outcomes among U.S. Adults

**DOI:** 10.3390/vaccines12050503

**Published:** 2024-05-07

**Authors:** Kimberly H. Nguyen, Cheyenne McChesney, Ruchi Patel, Robert A. Bednarczyk, Lavanya Vasudevan, Laura Corlin

**Affiliations:** 1 Hubert Department of Global Health, Rollins School of Public Health, Emory University, Atlanta, GA 30322, USA; 2 Department of Public Health and Community Medicine, School of Medicine, Tufts University, Boston, MA 02111, USA; 3 Department of Epidemiology, Rollins School of Public Health, Emory University, Atlanta, GA 30322, USA; 4 Emory Vaccine Center, Emory University, Atlanta, GA 30322, USA; 5 Department of Civil and Environmental Engineering, School of Engineering, Tufts University, Medford, MA 02155, USA

**Keywords:** COVID-19 vaccination, booster vaccination, vaccine hesitancy, vaccine confidence, outcomes, severity, long COVID, positive test, adults, United States

## Abstract

Understanding the association between booster vaccination and COVID-19 outcomes can help strengthen post-pandemic messaging and strategies to increase vaccination and reduce severe and long-term consequences of COVID-19. Using the Household Pulse Survey data collected from U.S. adults from 9 December 2022 to 13 February 2023 (n = 214,768), this study assessed the relationship between COVID-19 booster vaccination and COVID-19 outcomes (testing positive for COVID-19, moderate/severe COVID-19, and long COVID). Disparities were found in COVID-19 outcomes (e.g., testing positive for COVID-19, moderate/severe COVID-19, and long COVID) by sociodemographic characteristics, region of residence, food insecurity status, mental health status, disability status, and housing type. Receipt of a COVID-19 booster vaccination was negatively associated with testing positive for COVID-19 (aOR = 0.75, 95%CI: 0.72,0.79), having moderate/severe COVID-19 (aOR = 0.92, 95%CI: 0.88, 0.97), or having long COVID (aOR = 0.86 (0.80, 0.91)). Even among those who tested positive for COVID-19, those who received the booster vaccine were less likely to have moderate/severe COVID-19 and less likely to have long COVID. Communicating the benefits of COVID-19 booster vaccination, integrating vaccination in patient visits, and reducing access barriers can increase vaccination uptake and confidence for all individuals and protect them against the severe negative outcomes of COVID-19.

## 1. Introduction 

The COVID-19 pandemic contributed to high numbers of hospitalizations and deaths in the United States when it emerged in 2020 [1]. By the end of the year, the COVID-19 vaccine became available, but due to high demand, it was prioritized for those who were considered to be high risk early in the vaccination campaign, such as (1) healthcare personnel and residents of long-term care facilities; frontline essential workers and persons aged ≥75 years; persons aged 65–74 years; persons aged 18–64 years at high risk for severe COVID-19 illness because of underlying medical conditions; and other workers in essential and critical infrastructure sectors [2]. However, by April 2020, the vaccine was more widely available and recommended for all eligible adults ≥18 years [3]. By September 2022, COVID-19 bivalent booster vaccines became available to provide additional protection against COVID-19 and were recommended to all eligible children and adults 6 months and older [2]. While vaccination uptake steadily increased, there were disparities in coverage (by age, race/ethnicity, religion, geographical area, income level, and more), due to hesitancy and access barriers [4,5,6,7,8]. State and national vaccination campaigns focused on addressing misinformation, improving confidence in vaccines, and removing access barriers [9]. However, more effort is needed to highlight the importance of vaccines in protecting people against severe negative health outcomes from COVID-19. 

Despite a decreasing trend in COVID-19 deaths, hospitalizations, intensive care unit admissions, and high levels of population immunity to COVID-19 due to vaccination efforts and COVID-19 infection [10], the effects of COVID-19 remain, with many people still experiencing the devastating effects of post-COVID conditions, such as “long COVID” [11]. Following the COVID-19 pandemic, the priority of the U.S. Centers for Disease Control and Prevention (CDC) is to continue to monitor the impact of COVID-19 and the effectiveness of prevention and control strategies, such as vaccination [12]. While the effectiveness of COVID-19 vaccines against many variants has been well documented [13,14,15,16], the associations of COVID-19 booster vaccination and COVID-19 outcomes such as moderate/severe symptoms and long COVID are less known, which is important for targeted messaging and interventions to increase vaccine uptake and confidence. 

Despite the high uptake of COVID-19 vaccination of one or more doses, booster vaccination rates remain low in the U.S. (20.5% among adults as of May 2023) [17]. A COVID-19 booster vaccine is needed to help restore the protection that has waned since the previous vaccination by targeting new and emerging variants that are more transmissible and immune-evading [18]. Beginning on 1 September 2022, the CDC recommended that all eligible individuals receive the updated COVID-19 bivalent booster vaccine [2]. However, booster vaccination among U.S. adults has been low, due to reasons including the belief that it is not necessary or a lack of time to receive it [19,20]. 

Studies have documented disparities in COVID-19 primary series and booster vaccination, with gaps in coverage identified by age group, sex, race/ethnicity, educational attainment, and annual household income [19,20,21,22,23,24,25,26]. Furthermore, vulnerable populations such as those with food insecurity, mental health conditions, disabilities, and those living in transient homes such as mobile homes, recreational vehicles (RVs), and vans have also been shown to have lower vaccination coverage [27,28,29]. Those with the greatest disparities in coverage are more likely to have a greater risk of severe COVID-19 outcomes and death [16,30]. Understanding the association between booster vaccination and COVID-19 outcomes, overall and by groups which have been shown to have low booster coverage, can help inform post-pandemic strategies to increase vaccination and reduce severe and long-term consequences of COVID-19. 

## 2. Methods

### 2.1. Study Sample

As a nationally representative cross-sectional survey conducted by the U.S. Census Bureau, the Household Pulse Survey (HPS) collects data on adults aged ≥18 years to assess the impact of the COVD-19 pandemic on U.S. households. The survey design of the HPS has been described previously [31]. Non-institutionalized adults aged ≥18 years in the U.S. who were listed in the Census Bureau’s Master Address file and were matched to the Census Contact Frame were eligible to be selected for the survey. Selected participants were contacted via email and/or text and asked to complete a survey online using Qualtrics as a data collection platform. Data were collected from 9 to 19 December 2022 (response rate = 6.7%); 4 to 16 January 2023 (response rate = 6.4%); and 1 to 13 February 2023 (response rate = 7.0%) for a total sample size of 214,898 adults [32]. Respondents provided consent to participate in the HPS, and de-identified data are made publicly available on the U.S. Census website: https://www.census.gov/programs-surveys/household-pulse-survey/datasets.html (accessed on 1 March 2024). Per Emory University Institutional Review Board determination assessments, this study is not considered human subject research.

### 2.2. Measures

Testing positive for COVID-19 was assessed by the following question, “Have you ever tested positive for COVID-19 (using a rapid point-of-care test, self-test, or laboratory test) or been told by a doctor or other health care provider that you have or had COVID-19?” (yes/no). For people who reported that they had COVID-19, symptom severity was assessed by the following question: “How would you describe your coronavirus symptoms when they were at their worst?” Response options were “I had no symptoms/I had mild symptoms/I had moderate symptoms/I had severe symptoms”. Moderate/severe symptoms were combined into one category and no/mild were combined into another category. For people who reported that they had COVID-19, long COVID-19 was determined as anyone who responded yes to the question: “Did you have any symptoms lasting 3 months or longer that you did not have prior to having coronavirus or COVID-19? Long term symptoms may include tiredness or fatigue, difficulty thinking or concentrating, forgetfulness or memory problems (sometimes referred to as “brain fog”), difficulty breathing or shortness of breath, joint or muscle pain, fast-beating or pounding heart (also known as heart palpitations), chest pain, dizziness on standing, changes to your menstrual cycle, changes to taste/smell, or inability to exercise”. Those who responded yes to the question, “Do you have symptoms now?” were coded as having long COVID symptoms.

COVID-19 vaccination was assessed from the following variable: “Have you received a COVID-19 vaccine?” (yes/no). The date of the most recent COVID-19 primary series or booster vaccination was assessed by the following question: “How long ago was your most recent dose of the COVID-19 vaccine or booster?” Response options were “on or after 1 September 2022/Before 1 September 2022 but less than a year ago/more than a year ago”. People who stated that they were vaccinated and had their most recent dose on or after 1 September 2022 were considered to be boosted with the updated bivalent booster vaccine.

Socioeconomic variables assessed were respondent age group, sex, race/ethnicity, highest educational attainment, annual household income, health insurance status, and geographic region of residence. 

Food insecurity was assessed by the following question: “Getting enough food can also be a problem for some people. In the last 7 days, which of these statements best describes the food eaten in your household?” Those who responded with “Enough of the kinds of food (I/we) wanted to eat/Enough, but not always the kinds of food (I/we) wanted to eat” were categorized as food secure, while those who responded with “Sometimes not enough to eat/Often not enough to eat” were categorized as food insecure. 

Depression and anxiety were assessed using a validated two-item Patient Health Questionnaire (PHQ-2) and the two-item Generalized Anxiety Disorder (GAD-2) scale [33]. The questions on depression were the following: (1) “Over the last 2 weeks, how often have you been bothered by … having little interest or pleasure in doing things? and (2) … feeling down, depressed, or hopeless?” Questions on anxiety were the following: “Over the last 2 weeks, how often have you been bothered by the following problems (1) … feeling nervous, anxious, or on edge? and (2) … not being able to stop or control worrying?” For each question, the responses were “Would you say not at all (coded as 0), several days (coded as 1), more than half the days (coded as 2), or nearly every day (coded as 3)”. The two responses for each construct were summed. A score equal to three or greater on the PHQ-2 was categorized as symptoms of depression. A sum equal to three or greater on the GAD-2 was categorized as symptoms of anxiety. Adults who had either symptoms of anxiety or depression were categorized as having either disorder, while adults who had symptoms of neither anxiety nor depression were considered as not having either.

Disability was assessed as the following: (1) difficulty seeing, even when wearing glasses; (2) difficulty hearing, even when using a hearing aid; (3) difficulty remembering or concentrating; and (4) difficulty walking or climbing stairs [34]. The response options were (1) no difficulty, (2) some difficulty, (3) a lot of difficulty, and (4) cannot do at all. Those who answered “a lot of difficulty” or “cannot do at all” to any of the four categories of disability were categorized as having a disability, while those who answered “no difficulty” or “some difficulty” to all four questions were categorized as not having a disability.

Housing type was categorized as (1) single-family home (e.g., a detached one-family house), (2) condo or townhouse (e.g., a one-family house attached to one or more houses), (3) multi-unit housing (e.g., a building with 2 or more apartments), or (4) other (e.g., mobile home, boat, van, recreational vehicle (RV), or other).

### 2.3. Statistical Analysis

Sociodemographic characteristics were assessed for all adults ≥18 years. COVID-19 outcomes (positive test, long COVID, and moderate/severe COVID-19) were assessed overall and by each sociodemographic characteristic. We compared the proportion of people who reported COVID-19 outcomes by levels of sociodemographic characteristics. To examine the association between vaccination and COVID-19 outcomes, which were both dichotomous variables, logistic regression was used, which is consistent with other models used in the literature [35,36]. The association between COVID-19 vaccination (e.g., booster vaccination and date of most recent dose) and COVID-19 outcomes were assessed using separate multivariable logistic regression models adjusted for age, sex, race/ethnicity, educational attainment, income, health insurance, and geographic region. These variables were determined based on a review of the literature, which found these variables to be associated with COVID-19 vaccination and outcomes [30,37,38,39].The flow chart presented sample size estimates of the adults in the dataset by vaccination status, booster status, and COVID-19 outcomes. Results that are presented in the text of this manuscript are statistically significant at *p* < 0.05, and all analyses accounted for the survey design and weights to ensure a representative sample in Stata (version 18.0). 

## 3. Results

The sociodemographic characteristics of adults in the sample are provided in Table 1. Among respondents, 11.3% were food-insecure, 32.7% had anxiety or depression, and 15.6% had a disability. Furthermore, 19.4% lived in multi-unit housing, 7.1% lived in a townhouse or condo, and 5.6% lived in housing such as mobile homes, recreational vehicles, or vans. In the sample, 53.0% tested positive for COVID-19; of those, 53.0% had moderate/severe COVID-19 and 28.0% had long COVID (Table 2). The proportion who tested positive for COVID-19 was higher among people who were older than 65 years of age; females; those who identified as Hispanic compared to people who identified as NH White; people with incomes ≥USD 75,000 compared to people with lower incomes; people who were insured; people who lived in the Northeast compared to people in the Midwest or South; people who were food-secure; people with anxiety or depression; people without a disability; and people who lived in a single-family house compared to people in multi-unit housing or housing such as mobile homes, RVs, or vans. Largely in contrast to the trends for testing positive for COVID-19, the proportion of people who had COVID-19 and reported moderate or severe symptoms was higher among people who were older than 65 years of age; females; identified as Hispanic or NH other/multi-racial than among people who identified as NH White; people with incomes <USD 75,000 compared to people with higher incomes; people who resided in the West compared to the Northeast; people who were food-insecure; people who had anxiety or depression; people who had a disability; and people who resided in multi-unit housing compared to people in single-family housing. Also largely in contrast to the trends for testing positive for COVID-19, the proportion of people who had COVID-19 and reported long COVID symptoms was higher among people who were older than 65 years of age; females; people who identified as NH Black, Hispanic, or NH other/multi-race than among people who identified as NH White; people with a college degree or less education; people with incomes <USD 75,000 compared to people with higher incomes; people who were uninsured; people who lived outside the Northeast; people who were food-insecure; people with anxiety or depression; people with a disability; and people who lived in multi-unit housing or housing such as mobile homes, RVs, or vans compared to people in single-family housing.

**Figure 1:**
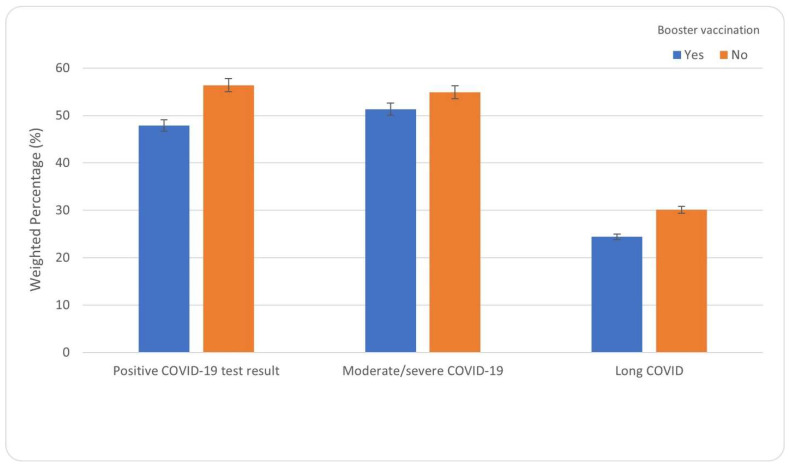
COVID-19 outcomes by booster vaccination status, Household Pulse Survey, 9 December 2022–13 February 2023.

Receipt of a COVID-19 booster vaccination was negatively associated with a positive test for COVID-19, moderate/severe COVID-19, and long COVID (Table 3, Figure 1). For example, those who received a booster vaccination were 0.75 times (95% confidence interval [CI]: 0.72, 0.79) as likely to test positive for COVID-19, 0.92 times (95%CI: 0.88, 0.97) as likely to have moderate/severe COVID-19, and 0.86 times as likely to have long COVID (95%CI: 0.80, 0.91) compared to those who did not receive a booster vaccination (Table 3). Recent doses were associated with having a protective effect on testing positive for COVID-19 (e.g., on or after 1 September 2022: adjusted odds ratio [aOR] = 0.74, 95%CI: 0.70, 0.78 compared to receiving a vaccination/booster more than a year ago. Receipt of a COVID-19 vaccination/booster on or after 1 September 2022 was also protective against long COVID (aOR = 0.86, 95%CI: 0.80, 0.93). 

**Figure 2:**
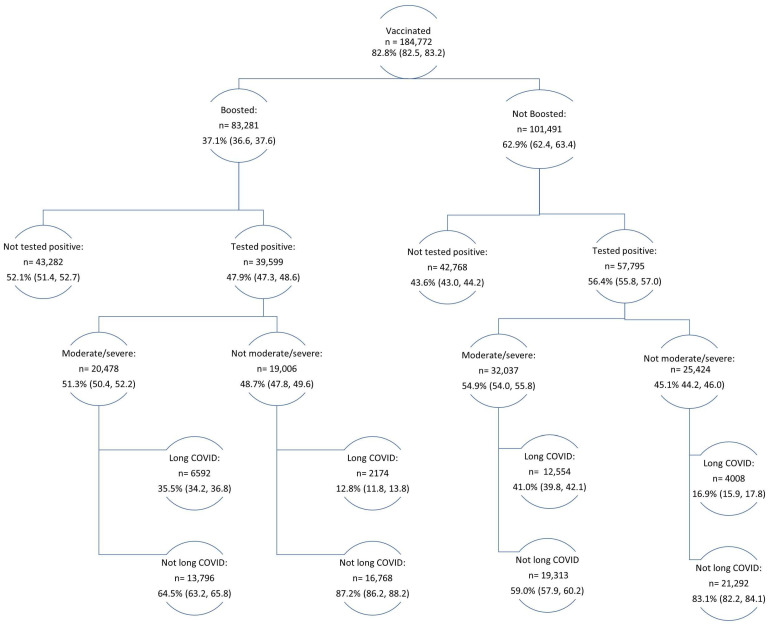
Flow chart of COVID-19 outcomes among boosted and non-boosted adults, Household Pulse Survey, 9 December 2022–13 February 2023.

In a flow chart showing the distribution of COVID-19 outcomes among all adults who were vaccinated, those who were boosted were less likely to test positive for COVID-19 (47.9%, 95%CI: 47.3, 48.6) than those who were not (56.4%, 95%CI: 55.8, 57.0) (Figure 2). Those who tested positive and were boosted were less likely to have moderate/severe COVID-19 (51.3%, 95%CI: 50.4, 52.2) than those who tested positive but did not get boosted (54.9%, 95%CI: 54.0, 55.8). Furthermore, people who had moderate/severe COVID-19 and were boosted were less likely to have long COVID (35.5%, 95%CI: 34.2, 36.8) than those who had moderate/severe COVID-19 but were not boosted (41.0%, 95%CI: 39.8, 42.1). Similarly, people who had less severe symptoms and were boosted were less likely to have long COVID (12.8%, 95%CI: 11.8, 13.8) than those who had less severe symptoms and were not boosted (16.9%, 95%CI: 15.9, 17.8).

## 4. Discussion

While more than one-half of people with COVID-19 had moderate/severe symptoms and more than one-fourth had long COVID as of February 2023, those who received booster vaccinations were less likely to have serious COVID-19 outcomes. This study found that booster vaccination was negatively associated with COVID-19 outcomes, such as positive tests, moderate/severe COVID-19, and long COVID. In addition, those who received more recent vaccinations were less likely to have a positive COVID-19 test, and those who received a vaccination after 1 September 2022 were less likely to have long COVID. For both people who experienced moderate or severe symptoms and for people who experienced less severe symptoms, having a booster vaccine was associated with lower likelihood of having long COVID symptoms. While this study cannot assess dose–response relationships due to the cross-sectional nature of the survey, these results suggest that receipt of recent boosters may be an indication of prior vaccination behavior and that the vaccination effects may be larger if more time-relevant data were available. These results underscore the importance of booster vaccination in preventing adverse COVID-19 outcomes, even among those who do not consider themselves to be high-risk. Being up to date with all recommended COVID-19 vaccines, particularly booster vaccinations, can protect against COVID-19 infection, moderate/severe COVID-19 symptoms, and the long-term effects of COVID-19. 

Many studies have already demonstrated the effectiveness of COVID-19 vaccines [13,40,41,42]. However, this study adds to the current literature by showing that being up to date with all eligible COVID-19 vaccinations, especially booster vaccinations, can provide additional protection. Investments in the development and testing of updated booster vaccines that are responsive to new and emerging COVID-19 virus strains are necessary to ensure the continued effectiveness of vaccinations. This is one of the first studies to examine disparities in the severity and length of COVID-19 symptoms by sociodemographic characteristics and high-risk conditions. This study found that adults who were self-reported Hispanic and NH other/multiple races and those who have food insecurity, anxiety or depression, or a disability were more likely than their respective counterparts to report having moderate/severe COVID-19 and long COVID. Additionally, in general, those with lower educational attainment and lower annual household income compared to their respective counterparts were less likely to test positive but more likely to have moderate/severe COVID-19 and long COVID. This suggests disparities in accessing vaccinations, diagnostic testing to identify all cases (including asymptomatic cases), and appropriate care when sick with COVID-19 to avoid worse outcomes. Similar patterns were found for those who were food-insecure, had a disability, or lived in housing such as mobile homes, RVs, and vans. These disparities underscore the need to increase equitable healthcare and resources to protect those who are most at risk. 

Increasing booster vaccination uptake and confidence is important among all populations for reducing severe COVID-19 outcomes, particularly among those with lower educational attainment or lower income and those with food insecurity, anxiety or depression, a disability, or housing such as mobile homes, RVs, and vans. Previous studies have shown that these populations have disparities in vaccination coverage and are at a higher risk for worse health outcomes [27,30,43,44,45,46]. The CDC also found that those who have a higher social vulnerability index, which measures indicators such as poverty, unemployment, housing burden, education, and health insurance, are likely to have lower COVID-19 vaccination rates [47]. These groups may also have lower intentions to be vaccinated, greater vaccine hesitancy, and/or greater barriers to access [48,49,50,51,52,53,54]. For these groups, targeted messaging and interventions to improve vaccination uptake and confidence and reduce barriers to access may be necessary to decrease health inequities and protect them from severe COVID-19 outcomes. 

The CDC recommends that all eligible individuals receive the updated bivalent booster vaccine to provide additional protection against new COVID-19 variants [2]. Healthcare providers can increase vaccination by offering and recommending vaccination as a default option during patient visits and integrating vaccination into medical practice procedures [9]. However, the quality and strength of vaccine recommendation may be influenced by the providers’ own vaccine hesitancy. Continuing medical education efforts could serve to communicate the most up-to-date evidence and vaccination guidelines to providers, especially as new booster vaccinations are developed and implemented. Guidance and advocacy by national provider networks may offer alternative avenues for reducing providers’ vaccine hesitancy. This and other publications highlight the need to increase access to vaccines and healthcare more broadly to reach sub-populations that are most likely to miss out on vaccines. Integrating COVID-19 booster vaccination in patient visits and medical practice procedures, and reducing transportation and access barriers, may help increase vaccination uptake and protect vulnerable sub-populations against the harmful effects of COVID-19.

### Limitations of this Study

The HPS is one of the largest, nationally representative surveys on COVID-19 vaccination in the U.S.; however, the data may be subject to several limitations. First, respondents might not be fully representative of the general U.S. adult population despite the sampling and weighting methodology that was conducted to ensure nationally representative data [31]. While the HPS may overestimate COVID-19 vaccination and booster coverage data compared to provider-reported vaccine administration data, the HPS estimates showed similar trends in coverage by sex, age group, and state compared to the provider-reported vaccine administration data [55,56]. Consequently, trends and disparities identified using the HPS study have been found to be consistent with those from the CDC’s provider-reported vaccination data [5,57]. Second, vaccination status and COVID-19 outcomes were self-reported and may have been subject to recall or social desirability bias. Furthermore, it is not certain that respondents received the updated bivalent booster vaccine, even though they reported that they received the vaccine after 1 September 2022. Third, cases may be asymptomatic and may be under-reported in this study. Fourth, access to testing was not measured; as a result, reporting not positive for COVID-19 may be due to less frequent or a lack of testing. Fifth, the HPS is a cross-sectional study so causality cannot be determined. Finally, response rates for telephone and web surveys are traditionally low, possibly due to the lack of perceived reward for responding, perceived cost of responding (e.g., time), and lack of trust that the rewards will outweigh the costs [58]. The HPS is no exception; it has a low response rate (<10%). However, the Census Bureau conducted a non-response bias and found that the survey weights mitigated most of this bias [59].

## 5. Conclusions

This study demonstrates the importance of being up to date with all eligible COVID-19 vaccinations, especially booster vaccinations, which were found to be associated with less likelihood of experiencing adverse COVID-19 outcomes. Increasing access to vaccines and early identification of cases and associated treatment to reduce symptom severity and length of illness, as well as improving vaccine confidence among all adults, especially those who are most at risk, may protect them against severe and long-term COVID-19 outcomes. Although the Public Health Emergency for COVID-19 has ended, ensuring that morbidity and mortality from COVID-19 continues to decline, and that everyone is protected against severe negative effects from COVID-19, is important for advancing the health of the nation.

## Figures and Tables

**Table 1:** Sociodemographic characteristics of adults, United States, Household Pulse Survey, 9 December 2022–13 February 2023.

	Unweighted N	Weighted % (95%CI)
**Overall**	214,898	
**Age group (years)**		
18–29	18,050	16.7 (16.5, 16.9)
30–39	40,543	18.7 (18.5, 18.8)
40–49	41,788	16.9 (16.7, 17.0)
50–64	59,385	25.9 (25.8, 26.1)
≥65	55,132	21.9 (21.8, 22.0)
**Sex**		
Male	92,049	48.7 (48.7, 48.7)
Female	122,849	51.3 (51.3, 51.3)
**Race/ethnicity**		
Non-Hispanic (NH) White	160,904	61.9 (61.9, 62.0)
NH Black	16,402	11.5 (11.5, 11.6)
Hispanic	19,061	17.3 (17.3, 17.4)
NH Asian	9958	5.2 (5.0, 5.3)
NH other/multiple	8573	4.0 (3.9, 4.2)
**Educational attainment**		
High school or less	31,145	37.9 (37.9, 37.9)
Some college or associate’s	67,652	29.4 (29.4, 29.4)
College graduate	60,970	18.0 (17.8, 18.1)
Above college graduate	55,131	14.7 (14.5, 14.8)
**Annual household income**		
<USD 35,000	31,984	18.1 (17.6, 18.5)
USD 35,000–USD 49,999	18,469	9.3 (9.0, 9.5)
USD 50,000–USD 74,999	29,088	13.2 (12.9, 13.5)
≥USD 75,000	96,177	35.4 (35.2, 35.7)
Did not report	39,180	24.0 (23.7, 24.4)
**Insurance status**		
Insured	179,249	93.0 (92.7, 93.2)
Not insured	7690	7.0 (6.8, 7.3)
**Region**		
Northeast	31,044	17.3 (17.3, 17.3)
Midwest	46,183	20.6 (20.6, 20.6)
West	67,661	23.7 (23.7, 23.7)
South	70,010	38.4 (38.4, 38.4)
**Food insecurity**		
Yes	14,917	11.3 (11.0, 11.6)
No	183,526	88.7 (88.4, 89.0)
**Anxiety or depression**		
Yes	53,547	32.7 (32.3, 33.1)
No	133,645	67.3 (66.9, 67.7)
**Have a disability**		
Yes	25,187	15.6 (15.3, 15.9)
No	158,094	84.4 (84.1, 84.7)
**Type of home**		
Single family	125,110	67.9 (67.4, 68.3)
Townhouse	14,058	7.2 (6.9, 7.4)
Multi-unit housing	33,581	19.4 (19.0, 19.7)
Mobile home, recreational vehicle, van, etc.	7741	5.6 (5.4, 5.8)

Note: All percentages are weighted. Abbreviations: CI = confidence interval.

**Table 2:** COVID-19 outcomes by sociodemographic characteristics, Household Pulse Survey, 9 December 2022–13 February 2023.

	Tested Positive for COVID		Moderate/Severe COVID		Long COVID	
	n	% (95%CI)	n	% (95%CI)	n	% (95%CI)
**Overall**	111,979	53.0 (52.6, 53.4)	59,695	53.0 (52.4, 53.6)	29,517	28.0 (27.4, 28.5)
**Age group (years)**						
18–29	10,884	59.9 (58.7, 61.0) *	6025	54.5 (52.6, 56.4) *	2739	26.9 (25.3, 28.5) *
30–39	25,025	60.4 (59.5, 61.2) *	13,383	53.4 (52.3, 54.6) *	6341	26.7 (25.7, 27.7) *
40–49	24,688	58.4 (57.6, 59.3) *	13,598	54.1 (52.9, 55.2) *	7041	31.1 (30.0, 32.2) *
50–64	30,123	50.8 (50.0, 51.6) *	16,500	53.7 (52.7, 54.8) *	8687	30.4 (29.5, 31.2) *
≥65 (reference)	21,259	40.0 (39.2, 40.9)	10,189	48.4 (47.5, 49.4)	4709	23.6 (22.5, 24.6)
**Sex**						
Male (reference)	46,270	51.0 (50.4, 51.7)	21,707	47.3 (46.3, 48.3)	9143	22.1 (21.3, 23.0)
Female	65,709	54.9 (54.4, 55.4) *	37,988	58.0 (57.4, 58.7) *	20,374	33.1 (32.4, 33.8) *
**Race/ethnicity**						
NH White (reference)	84,611	53.9 (53.4, 54.3)	44,945	52.5 (51.8, 53.2)	21,659	26.9 (26.4, 27.3)
NH Black	7162	42.5 (41.2, 43.8) *	3759	52.9 (51.1, 54.7)	2113	29.4 (27.6, 31.2) *
Hispanic	10,682	57.4 (56.3, 58.5) *	5901	55.1 (53.2, 57.0) *	3287	31.8 (30.2, 33.4) *
NH Asian	4932	51.2 (49.6, 52.8) *	2480	49.5 (47.4, 51.5) *	872	20.1 (18.0, 22.2) *
NH other/multiple	4592	53.3 (51.6, 55.0)	2610	55.6 (53.4, 57.8) *	1586	34.0 (31.7, 36.3) *
**Educational attainment**						
High school or less	14,167	48.1 (47.2, 49.0) *	7583	52.5 (51.1, 54.0)	4542	31.0 (29.8, 32.2) *
Some college or associate’s	34,312	54.1 (53.5, 54.7) *	19,178	55.1 (54.1, 56.0) *	11,362	31.7 (30.8, 32.5) *
College graduate	33,562	58.5 (57.9, 59.1) *	17,525	52.3 (51.4, 53.2) *	7500	22.7 (21.9, 23.4) *
Above college graduate (reference)	29,938	56.6 (56.0, 57.3)	15,409	50.9 (50.1, 51.7)	6113	21.1 (20.4, 21.9)
**Annual household income**						
<USD 35,000	13,405	43.7 (42.7, 44.7) *	8035	58.5 (57.0, 60.0) *	5250	38.4 (36.8, 40.0) *
USD 35,000–USD 49,999	8826	50.4 (48.7, 52.1) *	5063	56.0 (53.9, 58.2) *	2984	33.2 (31.3, 35.2) *
USD 50,000–USD 74,999	14,776	52.5 (51.5, 53.6) *	8074	53.7 (52.3, 55.2) *	4431	29.3 (27.7, 30.8) *
≥USD 75,000 (reference)	55,661	58.7 (58.2, 59.3)	28,617	51.0 (50.2, 51.8)	11,963	23.2 (22.6, 23.9)
Did not report	19,311	52.8 (51.8, 53.8) *	9906	51.2 (49.5, 52.8)	4889	26.4 (25.3, 27.5) *
**Insurance status**						
Insured (reference)	94,692	53.4 (53.0, 53.8)	50,640	53.1 (52.3, 53.8)	24,848	27.8 (27.2, 28.4)
Not insured	3562	46.9 (44.9, 48.9) *	2040	55.6 (52.7, 58.5)	1271	35.5 (32.7, 38.3) *
**Region**						
Northeast (reference)	17,341	55.8 (54.7, 56.9)	9174	52.0 (50.5, 53.5)	3975	24.7 (23.4, 25.9)
Midwest	23,816	52.0 (51.1, 52.9) *	12,342	52.1 (51.1, 53.1)	6489	27.9 (27.0, 28.9) *
West	34,834	54.5 (53.6, 55.4)	19,369	55.1 (53.8, 56.5) *	9297	28.5 (27.5, 29.5) *
South	35,988	51.3 (50.7, 52.0) *	18,810	52.6 (51.7, 53.4)	9756	29.2 (28.4, 30.1) *
**Food insecurity**						
Yes	7025	47.6 (46.2, 49.0) *	4440	59.5 (57.5, 61.4) *	3386	44.5 (42.4, 46.6) *
No (reference)	97,194	53.4 (53.0, 53.8)	51,426	52.6 (51.9, 53.2)	24,245	26.2 (25.6, 26.7)
**Anxiety or depression**						
Yes	28,601	54.1 (53.4, 54.8) *	18,050	61.9 (60.8, 63.0) *	11,866	40.8 (40.0, 41.7) *
No (reference)	69,850	52.4 (51.9, 52.9)	34,758	49.1 (48.2, 49.9)	14,332	22.0 (21.3, 22.7)
**Have a disability**						
Yes	83,939	50.5 (49.5, 51.5) *	7997	62.4 (60.9, 64.0) *	5895	45.9 (44.4, 47.3) *
No (reference)	12,477	53.4 (52.9, 53.9)	43,721	51.7 (51.0, 52.5)	19,721	25.3 (24.6, 25.9)
**Type of home**						
Single family (reference)	66,814	53.9 (53.4, 54.5)	35,153	52.3 (51.5, 53.0)	17,117	27.1 (26.4, 27.8)
Townhouse	7453	53.0 (51.2, 54.8)	4009	54.3 (52.3, 56.4)	1851	27.6 (25.8, 29.4)
Multi-unit housing	17,269	51.9 (50.9, 52.9) *	9880	57.1 (55.6, 58.5) *	4835	29.7 (28.3, 31.1) *
Mobile home, RV, van, etc.	3499	45.8 (43.9, 47.7) *	1975	53.9 (50.9, 56.9)	1434	40.6 (38.0, 43.2) *

Note: All percentages are weighted. Abbreviations: CI = confidence interval. * Significant difference in proportion of COVID-19 outcome (e.g., long COVID) among people with that level of a characteristic (e.g., age between 18 and 29 years) compared to people with the reference level of that characteristic (e.g., 65+ years).

**Table 3:** Association between receipt of COVID-19 booster vaccination and COVID-19 outcomes, Household Pulse Survey, 9 December 2022–13 February 2023.

	Tested Positive		Moderate/Severe		Long COVID	
	% (95%CI)	aOR (95% CI) ^a^	% (95%CI)	aOR (95% CI) ^a^	% (95%CI)	aOR (95% CI) ^a^
**Receipt of booster vaccination among vaccinated**						
Yes	47.9 (47.3, 48.6)	0.75 (0.72, 0.79)	51.3 (50.4, 52.2)	0.92 (0.88, 0.97)	24.4 (23.6, 25.2)	0.86 (0.80, 0.91)
No	56.4 (55.8, 57.0)	1	54.9 (54.0, 55.8)	1	30.1 (29.3, 30.9)	
**Date of most recent dose**						
On or after 1 September 2022	47.9 (47.3, 48.6)	0.74 (0.70, 0.78)	51.3 (50.4, 52.2)	0.97 (0.91, 1.03)	24.4 (23.6, 25.2)	0.86 (0.80, 0.93)
Before 1 September 2022 but less than a year ago	55.0 (54.2, 55.9)	0.96 (0.90, 1.01)	55.9 (54.6, 57.1)	1.10 (1.03, 1.19)	30.4 (29.4, 31.3)	1.01 (0.95, 1.08)
More than a year ago (reference)	57.9 (57.0, 58.8)	1	53.9 (52.8, 55.0)	1	29.8 (28.7, 30.9)	1

Note: All percentages are weighted. Abbreviations: aOR = adjusted odds ratio, CI = confidence interval. ^a^ Adjusted for age, sex, race/ethnicity, educational attainment, income, health insurance, and geographic region.

## Data Availability

The data that support the findings of this study are openly available at https://www.census.gov/programs-surveys/household-pulse-survey/datasets.html (accessed on 1 March 2024).
